# Optogenetics Sheds Light on Brown and Beige Adipocytes

**DOI:** 10.33696/signaling.4.105

**Published:** 2023

**Authors:** Aaron Clifford Brown

**Affiliations:** 1 MaineHealth Institute for Research, 81 Research Drive, Scarborough, ME 04074, USA; 2 School of Biomedical Sciences and Engineering, The University of Maine, Orono, Maine 04469, USA; 3 Tufts University School of Medicine, 145 Harrison Ave, Boston, MA 02111, USA

**Keywords:** Brown adipocytes, Beige adipocytes, Thermogenesis, Optogenetics, Obesity, Type 2 diabetes, UCP1, bPAC, ChR2, Calcium signaling

## Abstract

Excessive food intake leads to lipid accumulation in white adipose tissue, triggering inflammation, cellular stress, insulin resistance, and metabolic syndrome. In contrast, the dynamic energy expenditure and heat generation of brown and beige adipose tissue, driven by specialized mitochondria, render it an appealing candidate for therapeutic strategies aimed at addressing metabolic disorders. This review examines the therapeutic potential of brown and beige adipocytes for obesity and metabolic disorders, focusing on recent studies that employ optogenetics for thermogenesis control in these cells. The findings delve into the mechanisms underlying UCP1-dependent and UCP1-independent thermogenesis and how optogenetic approaches can be used to precisely modulate energy expenditure and induce thermogenesis. The convergence of adipocyte biology and optogenetics presents an exciting frontier in combating metabolic disorders and advancing our understanding of cellular regulation and energy balance.

## Unlocking the Therapeutic Potential of Brown and Beige Adipose Tissues

Extended periods of excessive food intake give rise to the accumulation of lipids within white adipose tissue (WAT), paving the way for the onset of inflammation, cellular stress, insulin resistance, and ultimately, metabolic syndrome [1]. In contrast, brown adipose tissue activation (BAT) exhibits a positive correlation with increased energy expenditure and reduced vulnerability to metabolic syndrome and cardiometabolic disorders, rendering it an enticing tissue for therapeutic intervention [2–5]. Notably, brown adipocytes possess abundant, highly specialized mitochondria that expend energy to generate heat, facilitating the maintenance of core body temperature via a process recognized as non-shivering thermogenesis [6].

BAT assumes its anatomical configuration during embryonic development, primarily located within distinct regions such as the interscapular, cervical-supraclavicular, and paravertebral zones in both rodents and human subjects [7,8]. Within these precise locations, BAT undergoes a remarkable process of extensive innervation and vascularization, becoming intricately intertwined with neural and blood vessel networks. The activation of BAT is predominantly triggered by the cold-induced release of norepinephrine originating from sympathetic nerve terminals, which subsequently binds to β-adrenergic surface receptors located on brown adipocytes [9]. Activation of β-adrenergic receptors sets in motion a cascade that initiates nutrient combustion. This entails: (1) the uptake of glucose and fatty acids/lipids from the bloodstream, coupled with the lipolysis of lipids derived from intracellular multilocular lipid droplets [10,11]; (2) the liberation of free fatty acids, priming them for subsequent β-oxidation; and (3) the creation of a proton gradient within the inner mitochondrial membrane, thereby activating the electron transport chain to facilitate ATP production [11]. Brown adipocytes exhibit a distinctive trait wherein β-adrenergic activation induces the transcription of uncoupling protein-1 (UCP1). This protein, situated on the inner mitochondrial membrane, functions to dissipate the electrochemical gradient established within the mitochondria, diverting it away from ATP synthesis. Instead, this redirection releases protons back into the mitochondrial matrix, yielding energy in the form of heat production [2]. This release in heat helps maintain core body temperature (euthermia) by warming the blood during exposure to cold temperatures.

Apart from brown adipocytes, a developmentally distinct subset of fat cells known as beige adipocytes, located within subcutaneous WAT, can experience temperature-triggered transformations from a white (UCP1−) to a brown-like (UCP1+) state [12]. Through β-adrenergic stimulation, beige adipocytes can actively participate in sustaining body temperature and enhancing resistance against obesity [13]. Furthermore, beige adipocytes are able to undergo UCP1-independent signaling, which can be mediated by futile Ca^2+^ signaling, a unique and finely orchestrated intracellular calcium (Ca^2+^) flux that culminates in thermogenesis [14].

Unfortunately, attempts to manipulate obesity and diabetes in humans via drug-induced activation of β-adrenergic receptors have yielded modest outcomes, as this approach bears the potential of elevating the risk of cardiovascular disease [15–17]. An alternative approach to chemical modulation of cell signaling is optogenetics, a state-of-the-art technique that uses light-sensitive proteins to control specific cellular processes in genetically modified cells that can be used for precise temporal, spatial and reversable control of signaling.

## Multifaceted Applications of Optogenetics

Optogenetics integrates genetic manipulation with optical control, allowing for unparalleled precision in regulating cellular activity across both time and space. Originally conceived to manipulate neurons, its applications now extend far beyond, encompassing a wide range of cell types. This groundbreaking technique has two essential components: light-sensitive proteins and genetic engineering. The pivotal breakthrough in this field came with the identification and utilization of microbial opsin proteins, such as channelrhodopsin and halorhodopsin, which are responsive to specific wavelengths of light [18]. These opsin genes can be introduced into target cell genomes through viral vectors or transgenic approaches. Once expressed, opsins can be used to either stimulate or suppress cellular activity in response to light. Channelrhodopsin was originally discovered in certain types of green algae and when it is genetically expressed in target neurons, exposure to light causes the protein to open ion channels in the neuron’s membrane, allowing positively charged ions to flow into the neuron [19,20]. This influx of ions triggers neuronal depolarization, leading to the generation of action potentials and neuron activation. Halorhodopsin, derived from halophilic archaebacteria, serves a complementary function to channelrhodopsin and can hyperpolarize neurons, effectively inhibiting their activity [21,22]. Halorhodopsin inhibits neuronal activity by pumping chloride ions into the neuron when exposed to light. This influx of negatively charged chloride ions hyperpolarizes the neuron’s membrane potential, making it more difficult for the neuron to reach the threshold required for firing action potentials. As a result, neuronal firing is suppressed, and the neuron becomes less responsive to incoming signals. Together these proteins and their derivatives have provided a platform for precise control of neural function via optogenetics.

Optogenetics has had a profound impact on neuroscience. In the realm of basic research, it has empowered scientists to disentangle the functional roles of specific neuronal populations within intricate networks. By introducing light-sensitive proteins into neurons, scientists can precisely activate or inhibit these cells, leading to insights into neural circuitry and behavior, as well as neurological disorders such as Parkinson’s disease, epilepsy, and depression [23–25]. Optogenetics has also been used to study the neural circuits governing memory formation and learning processes [26]. Through the manipulation of neurons implicated in memory tasks, researchers have discovered new pathways and potential therapeutic targets for conditions like Alzheimer’s disease [27]. In the field of vision, optogenetics holds potential for restoring sight to individuals afflicted by degenerative eye diseases [28]. By rendering remaining retinal cells light-sensitive, this technique may provide solutions for conditions like retinitis pigmentosa [29].

Despite its role in revolutionizing neuroscience, optogenetics is not confined solely to neurons. In recent years, researchers have harnessed the technique to manipulate a diverse array of non-neuronal cells. Optogenetics has been used to manipulate immune cells, opening new avenues for controlling immune responses and advancing cancer immunotherapy [30]. In cardiac cells, the use of optogenetics allows precise control over heart rhythm, which could pave the way for therapies for arrhythmias and other heart-related conditions [31]. The expansion of optogenetic techniques into non-neuronal domains holds great promise for advancing our understanding of various biological processes and pioneering innovative therapeutic strategies.

Within the context of brown and beige adipocytes, optogenetics can be used to manipulate thermogenic activity and explore thermogenesis regulation. In this concise review, we spotlight three noteworthy studies that have harnessed optogenetics to activate thermogenesis in adipocytes and discuss the implications of this for therapeutic intervention in the ongoing battle against obesity-related diseases.

## Optogenetic Activation of BAT through Sympathetic Neuromodulation via ChR2

**Figure 1.**
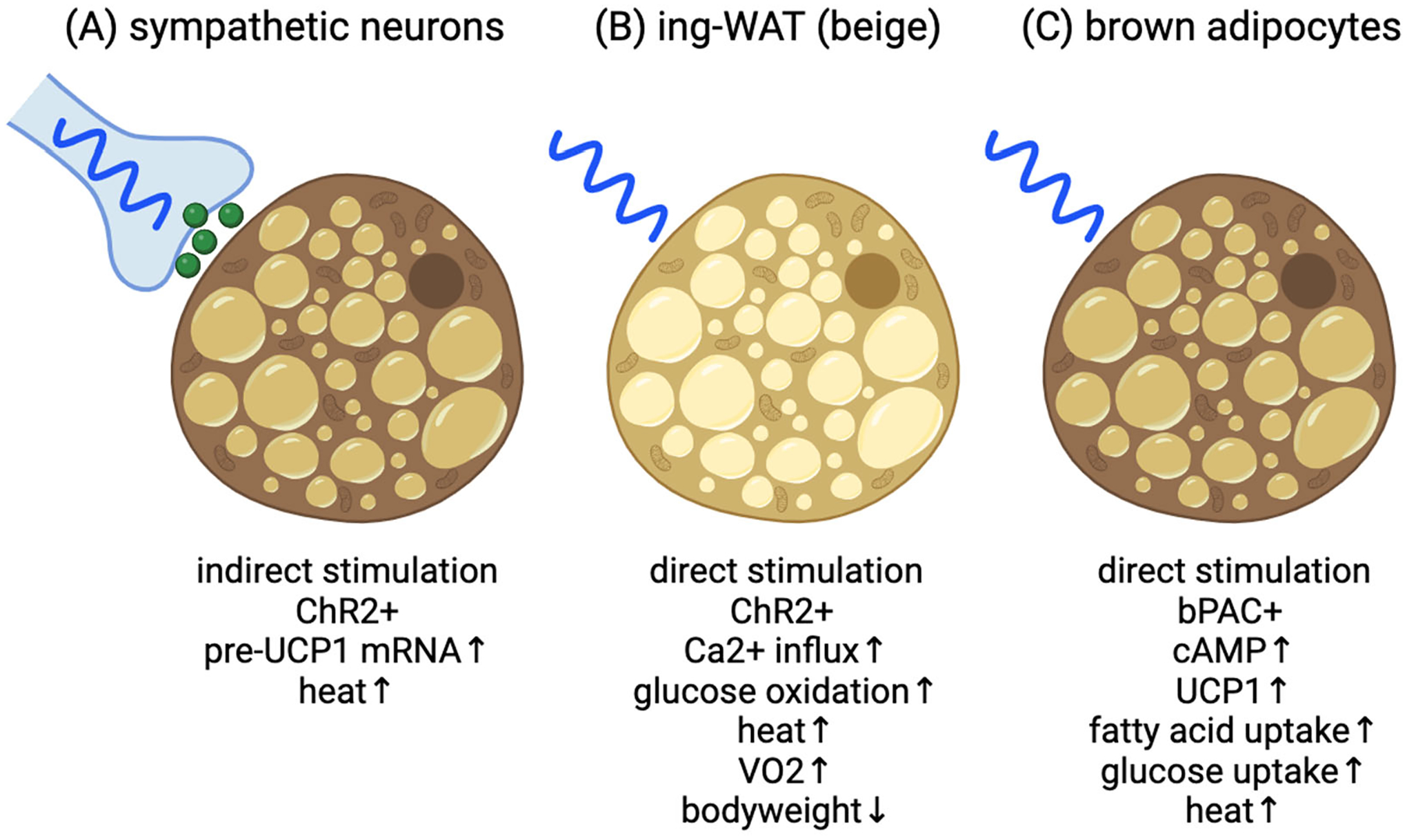
Current models for optogenetic activation for adipocyte thermogenesis. **(A)** Blue light stimulation of ChR2+ sympathetic neurons adjacent to brown adipose tissue results in increased BAT thermogenesis and whole-body temperature. Presumptive norepinephrine secretion indicated by green spheres. **(B)** Blue light stimulation of ChR2+ inguinal white adipose tissue (ing-WAT) results in UCP1-independent thermogenesis via Ca^2+^ signaling leading to increased whole-body oxygen consumption rate and weight loss. **(C)** Blue light stimulation of bPAC+ brown adipocytes increase cAMP signaling leading to increased UCP1 expression, fuel uptake and heat production. Image created at BioRender.com.

In 2019, the Bartolomucci Laboratory (Lyons et. al. [32]) published a study exploring whether selective optogenetic stimulation of sympathetic nerves innervating BAT could induce thermogenesis (Figure 1A). Two approaches were employed. The first involved modifying adeno-associated-AAV6 virus to express the blue light-sensitive cation channel ChR2 fused with enhanced yellow fluorescent protein (EYFP), controlled by the pan-neuronal human synapsin-1 promoter. Injecting this engineered AAV6 into BAT of mice led to successful transduction of TH-positive sympathetic nerves, confirming expression in the right locations. A custom made blue light LED (455nm) with a thermoprobe to allow for temperature measurements was implanted beneath the BAT through an incision on the back. Subsequently, AAV6-ChR2-EYFP-injected mice underwent a 30-minute blue LED light stimulation, resulting in significant increases in both BAT and core body temperature (as determined by rectal probe), distinct from AAV6-EYFP controls. This protocol also notably elevated transcriptional activation of *Ucp1* pre-mRNA in ChR2-expressing mice compared to controls. Genetic validation followed as the second approach, employing TH-Cre X Rosa26-LSL-ChR2-YFP mice (ChR2TH-Cre+ or ChR2TH-Cre−). ChR2TH-Cre+ mice showed BAT thermogenesis upon optogenetic stimulation, evidenced by both BAT and core body temperature elevation, whereas ChR2TH-Cre− did not. The temperature elevation persisted after the LED was switched off for at least an additional 30 minutes. Moreover, increased *Ucp1* pre-mRNA expression was observed in ChR2TH-Cre+ mice. These results demonstrate that optogenetic stimulation of sympathetic nerves is sufficient to induce BAT thermogenesis, indicated by increases in temperature and *Ucp1* pre-mRNA. This presumably would occur due to release of norepinephrine from sympathetic nerves terminals adjacent to the brown adipocytes, however, norepinephrine secretion was not analyzed during the study. Overall, this approach holds promise for addressing obesity and diabetes through peripheral neuromodulation.

## Stimulation of UCP1-independent Fat Thermogenesis in Subcutaneous Adipose Tissue Using Wireless Optogenetics

Thermogenesis in adipose tissue can be stimulated by cold exposure, leading to the release of norepinephrine from the sympathetic nervous system. Norepinephrine activates the β3-adrenergic receptor (β3-AR) and its downstream signaling pathway, which triggers thermogenesis through various mechanisms, including expression and activation of UCP1. While efforts have been made to develop β3-AR agonists to promote adipose tissue thermogenesis as an anti-obesity treatment, these drugs often come with cardiovascular side effects such as high blood pressure [15,16]. A study by the Kajimura Laboratory (Tajima et. al. [33]) explored an alternative pathway to stimulate fat thermogenesis that may bypass these cardiovascular risks (Figure 1B). One such pathway is non-canonical UCP1-independent thermogenesis, previously identified in beige fat, which involves ATP-dependent calcium (Ca^2+^) futile cycling [14]. This particular study used a chemical stabilizer (S107) to enhance Ca^2+^ cycling and stimulate thermogenesis in mice, but couldn’t confirm whether other tissues besides adipose tissue played a role. To address this limitation, the researchers developed a wireless optogenetics device that could be implanted adjacent to the subcutaneous adipose tissue of mice. The wireless optogenetics device consists of a small power-harvesting coil connected to a receiving circuit and a blue micro-LED to activate beige adipocytes expressing channelrhodopsin 2 (ChR2). The study optimized the wireless device’s power transfer efficiency and ChR2 activation, identifying that a 5-millisecond pulse width and a 10 Hz frequency were ideal for stimulating intracellular Ca^2+^ influx in cultured beige adipocytes and without generating excessive heat when the device was implanted subcutaneously.

Activation of ChR2 in cultured beige adipocytes with blue light led to intracellular Ca^2+^influx, and this was mediated by L-type voltage-dependent Ca^2+^ channels. Inhibition of these channels with isradipine blocked the Ca^2+^ influx, confirming their involvement. Additionally, the study found that Ca^2+^ release from the endoplasmic reticulum via Ryanodine Receptor 2 and inositol 1,4,5-trisphosphate receptors was necessary for light-induced Ca^2+^ cycling. Furthermore, the activation of Ca^2+^ cycling in beige adipocytes was sufficient to stimulate thermogenesis, increase cellular oxygen consumption, and enhance glucose oxidation. Using *Ucp1* null beige adipocytes, the researchers confirmed that this effect was UCP1-independent and primarily relied on SERCA2-mediated Ca^2+^ cycling.

Moving from cellular experiments to *in vivo* studies, the wireless optogenetics device was implanted under the skin adjacent to subcutaneous adipose tissue of adiponectin-Cre conditional mice expressing ChR2 specifically in adipocytes. Light activation of ChR2 in these mice led to a significant increase in adipose tissue temperature, demonstrating that the device could stimulate thermogenesis *in vivo*, even without cold exposure. The study also investigated the effects of light-induced thermogenesis on whole-body energy expenditure. Mice with the implanted device showed a significant increase in whole-body oxygen consumption without altering their physical activity, indicating enhanced thermogenesis and energy expenditure. Notably, this increase in energy expenditure was comparable to that achieved through pharmacological activation of β3-AR with CL316,243. Finally, the researchers tested whether optogenetic stimulation of thermogenesis could protect mice from diet-induced obesity. Mice with the wireless optogenetics device implanted in their adipose tissue were exposed to a high-fat diet (HFD). Light activation of thermogenesis for 10 minutes per day (10-Hz frequency and 5-ms pulse width) in these mice led to reduced body weight gain, specifically due to decreased fat mass, without affecting lean mass. The inguinal WAT of these mice was smaller, containing smaller adipocytes, and displayed no significant changes in inflammation or fibrosis markers.

In summary, this study demonstrates that wireless optogenetic stimulation of thermogenesis in adipose tissue can effectively increase energy expenditure and protect against diet-induced obesity in mice. The approach primarily relies on Ca^2+^ cycling through SERCA2 and represents a promising avenue for developing safe and effective treatments for obesity and related metabolic disorders.

## Stimulation of UCP1-dependent Thermogenesis in Brown Adipocytes Using Optogenetics

**Figure 2.**
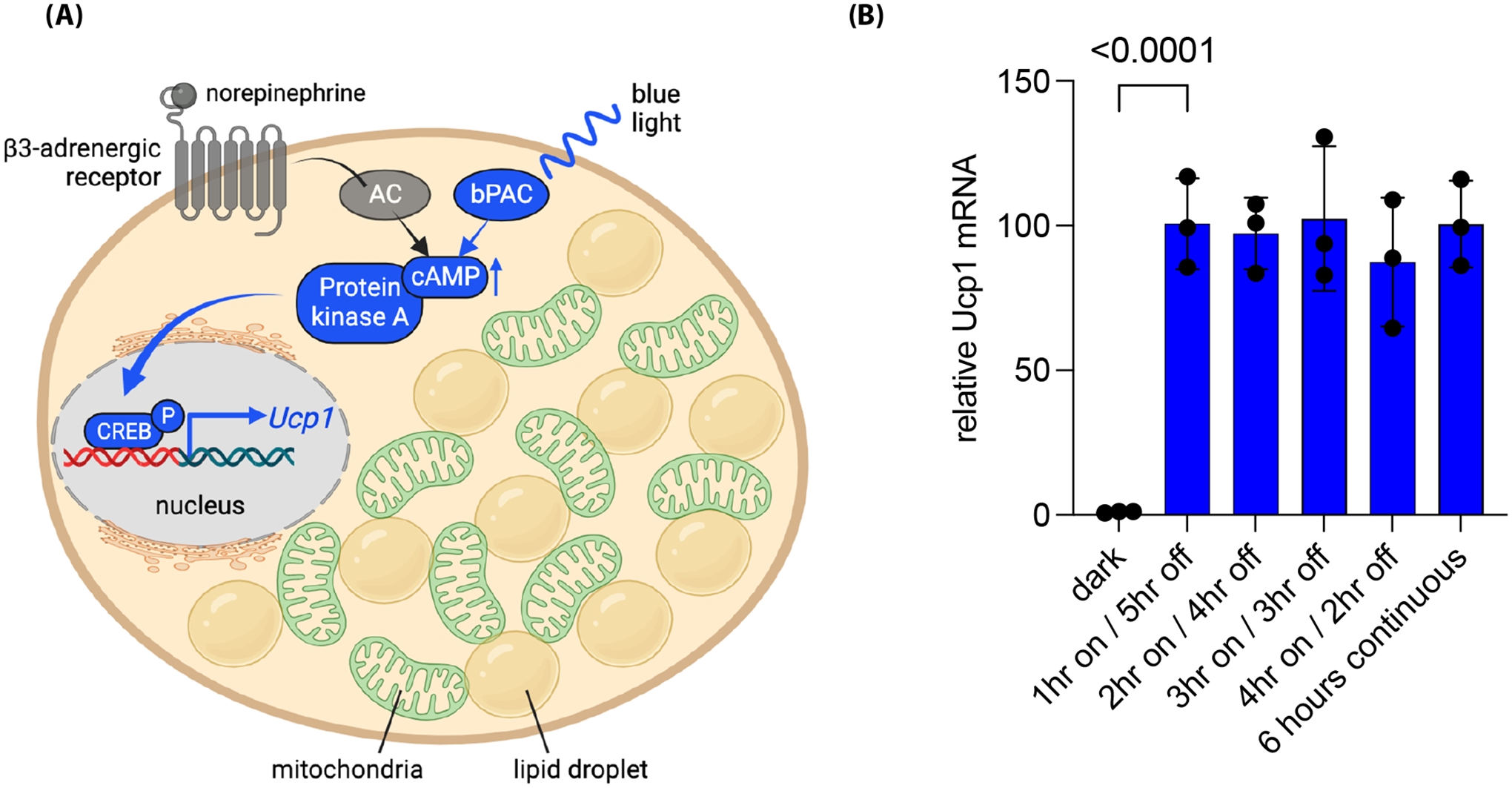
Blue light stimulation of bPAC leads to increased expression of *Ucp1* in beige adipocytes. **(A)** Illustrative depiction of the process by which optogenetic stimulation of bPAC results in elevated *Ucp1* expression in beige or brown adipocytes. Blue light initiates the activation of cytoplasmic bPAC, bypassing the necessity for norepinephrine-induced adipocyte stimulation via the β3-adrenergic receptor and its subsequent activation of endogenous adenylyl cyclase (AC). The bPAC signaling cascade elevates intracellular cAMP levels, leading to the activation of protein kinase A. This, in turn, facilitates the phosphorylation of CREB and its translocation into the nucleus, where it collaborates with other thermogenic transcription factors (not shown) to boost *Ucp1* transcription. **(B)** SV40 large T antigen immortalized preadipocytes derived from mouse inguinal adipose tissue (Kerafast, Inc. Cat# EVC005) were differentiated into mature beige adipocytes for 9 days and pulsed with blue light at time zero for the indicated times and harvested at 6 hours as previously described [40,54]. qPCR data indicates that beige adipocytes induce and maintain *Ucp1* expression for at least 5 hours with a single 1-hour pulse of blue light (470nm). Data are normalized to β-actin expression. Error bars represent ± standard deviation with one-way ANOVA analysis p< 0.0001 indicated. Figure A created at BioRender.com and modified from Doucette et al. [40].

While thermogenic adipose tissue inherently possesses anti-obesity attributes, our understanding of the regulatory mechanisms underlying UCP1-dependent thermogenesis remains incomplete. The control of UCP1 encompasses various tiers, spanning from its transcriptional initiation to the turnover of its mRNA and protein. Furthermore, its activation takes place within the inner mitochondrial membrane, where it remains inactive until β-adrenergic activation [34,35]. To initiate the transcription process, β-adrenergic receptors linked with Gs-alpha subunits first trigger the activation of membrane-associated adenylyl cyclases, prompting a surge in intracellular cAMP levels [36] (Figure 2A). This heightened cAMP concentration subsequently triggers the activation of cAMP-dependent protein kinase A (PKA), leading to lipolysis and consequent release of free fatty acids—an essential prerequisite for activation of UCP1-mediated proton leakage [37]. PKA’s influence extends further to the phosphorylation of CREB and activation of a cluster of thermogenic transcription factors. Ultimately, this signaling cascade causes increased mitochondrial biogenesis, amplified electron transport chain activity, and the initiation of *Ucp1* transcription (Figure 2A), which collectively allow thermogenesis to take place [38].

To determine if brown adipocytes could be genetically modified for light inducible activation of UCP1-dependent thermogenesis, our laboratory took advantage of a bacterial photoactivatable adenylyl cyclase (bPAC), previously cloned from the soil bacterium *Beggiatoa* for chemical free stimulation of adenylyl cyclase [39,40]. bPAC is made up of two parts: an N-terminal BLUF domain (blue light sensor using flavin adenine dinucleotide) and a C-terminal class IIIb adenylyl cyclase. The active site of these cyclases is created by two cyclase domains working together as a homodimer [41]. Upon stimulation with blue light, bPAC has been shown to increase cAMP levels and alter cellular physiology in cells from numerous species including *Escherichia coli*, *Drosophila melanogaster* (nervous system), *Xenopus laevis* (oocytes), *Mus musculus* (sperm), *Rattus norvegicus* (pyramidal neurons) and *Homo sapiens* (HEK-293T cells) [39,42,43].

In our study, brown preadipocytes derived from ThermoMouse [44] expressing the blue-light-sensitive bPAC protein were generated via lentiviral transduction and used to investigate the effects of blue light stimulation on cAMP signaling and gene expression related to brown adipocyte function (Figures 1C and 2A) (Doucette et. al. [40]). Blue light stimulation, achieved through a custom microplate photo irradiation system with blue LEDs, was used to activate brown preadipocytes [45]. We found that short blue light pulses (6 seconds to 1 minute @470nm) led to significant increases in cAMP levels, comparable to the effects of forskolin, a known stimulator of endogenous adenylyl cyclase. Importantly, this effect was specific to cAMP signaling, as there was no observed cGMP production after blue light stimulation. Blue light also triggered the phosphorylation of CREB and the expression of the immediate-early CREB target gene *Nr4a3*, indicating that bPAC activation could lead to transcriptional responses. We also explored the effects of different patterns of blue light stimulation on *Ucp1* transcription in brown adipocytes. Our results show that continuous, uninterrupted blue light exposure (up to 6 hours) led to the highest levels of *Ucp1* expression. These results were comparable to brown adipocytes treated with forskolin, suggesting that bPAC is as efficient as endogenous adenylyl cyclases in stimulating *Ucp1* expression. Furthermore, the expression of genes related to mitochondrial biology, including those involved in mitochondrial biogenesis, ATP synthesis, electron transport, and mitophagy were increased, suggesting that light-induced stimulation of cAMP signaling promoted mitochondrial function in brown adipocytes. In cell culture, brown adipocytes typically lose UCP1 expression over time in the absence of stimuli activating downstream cAMP signaling. We found that intermittent optogenetic stimulation with daily blue light pulses of 30 minutes could prevent loss of UCP1 expression over time. We also examined the impact of blue light stimulation on fuel uptake and thermogenesis in brown adipocytes. Activation of bPAC with blue light led to increased fatty acid and glucose uptake and microcalorimetry measurements demonstrated that bPAC+ brown adipocytes produced significantly more heat (≈4-fold) in response to blue light compared to control cells. We also confirmed the UCP1-dependent nature of bPAC-induced thermogenesis using short hairpin RNA to target *Ucp1* for degradation, which resulted in a lack of light-induced heat production. Overall, these findings suggest that blue light stimulation of bPAC can promote UCP1-dependent thermogenesis in brown adipocytes, providing a strategy for regulating brown adipocyte activity. Next steps will be to generate *in vivo* mouse models to test this system for increased energy expenditure and weight loss.

One lingering question regarding the use of bPAC-expressing brown adipocytes for weight loss pertains to the kinetics of brown adipocyte activation. This is particularly relevant as the induction of thermogenesis in bPAC+ brown adipocytes require longer pulses of blue light compared to the UCP1-independent ChR2 studies mentioned above. Prolonged exposure to blue light pulses to sustain *Ucp1* expression could potentially lead to excessive heat generation via a wireless blue light LED, compromising cellular behavior or promoting tissue damage. An alternative hypothesis revolves around the idea that brown adipocytes might need continuous cAMP signaling to uphold thermogenesis in comparison to beige adipocytes. Recent experiments conducted in our laboratory on beige adipocytes transduced with bPAC have shed some light on this matter. One experiment revealed that *Ucp1* expression reached its peak at the one-hour mark and remained elevated for an additional five hours in the absence of light (Figure 2B). This level of expression was similar to what was observed with six hours of uninterrupted blue light exposure in the brown adipocytes. This finding raises the possibility that beige adipocytes may have a lower activation threshold for UCP1-dependent thermogenesis through bPAC stimulation, although this hypothesis will require further validation. Future investigations will be performed to determine the minimal amount of blue light (<1 hour) required to maintain *Ucp1* expression and sustain thermogenesis in beige adipocytes effectively. Additionally, exploring further modifications of PACs or discovering new PACs that exhibit heightened sensitivity may hold promise for achieving a more rapid induction of UCP1-dependent thermogenesis.

## Navigating the Challenges of Optogenetics-based Therapies and Obesity Management

Technical challenges must be overcome to transition optogenetics from a research tool to a viable therapeutic approach for human diseases. Currently, only four early-phase clinical trials are in progress, all focused on treating retinitis pigmentosa [46]. The available data, though limited, indicates favorable tolerability of the optogenetic gene therapy [46,47]. The accessibility of the eye to light makes optogenetics promising for visual conditions, but trials for other conditions may require years for the development of safe implantable light sources. The success of optogenetics in humans depends on optimizing gene delivery methods, particularly viral vectors like adeno-associated viruses (AAVs), while addressing concerns about immune reactions, delivery precision, and safety [48]. One primary challenge is delivering light in a manner suitable for human-scale tissue volumes. Enhancements in optogenetic tools, including increased sensitivity to light and the use of far-red light to minimize tissue damage, offer potential solutions [48]. Optogenetic techniques may have unintended off-target effects on neighboring cells or tissues, and achieving precise targeting of specific cells remains an ongoing challenge. Blue light, commonly used for optogenetic activation, has limited tissue penetration, and requires transplantation of blue-light transmitters to target internal tissues [33,49]. The development of far-red optogenetic proteins may eliminate the need for wireless transmitters since an external source of red light can penetrate deep into tissues [48,50]. However, the long-term stability and viability of optogenetic tools within the body are not understood, posing a concern for therapeutic applications. Manipulating cellular processes can lead to unpredictable consequences, and the potential side effects and unintended outcomes must be addressed. The transition from experimental use in animals to clinical applications in humans will involve regulatory challenges, safety testing, and ethical considerations, making clinical translation a complex and time-consuming process [48]. As optogenetic methods advance, therapeutic applications should gradually make their way into clinical trials.

The biology of adipose tissue could help circumvent numerous challenges associated with optogenetic therapies in other organ systems and tissues. Unlike the targeting of native cells, such as specific neuronal subsets in the spinal cord or brain, brown and beige adipose tissue has the potential to be transplanted to facilitate weight loss. Patient-specific beige adipose tissue can be generated in the laboratory from adipose-derived stem cells (ADSCs) that are obtainable through tissue biopsy or liposuction [51,52]. Transgenic modification of ADSCs with optogenetic genes and their subsequent differentiation into beige adipocytes could be performed in the laboratory prior to reintroduction into patients. Studies confirm the successful vascularization of human-derived beige adipose tissue, which has been shown to enhance systemic glucose tolerance when transplanted in mice [53]. One drawback to the use of ADSCs derived from lipoaspirates is that they might have limited expansion and differentiation capacity, especially when derived from patients with metabolic disease [54]. Patient-specific induced pluripotent stem cells (iPSCs) provide an alternative source for brown and beige adipocytes, ensuring a replenishable, unlimited supply of material for weight loss applications [54–56]. Furthermore, recently developed non-immunogenic iPSCs may mitigate concerns regarding immunogenicity related to the display of optogenetic peptides on transplanted cells, while eliminating the need for patient-specific derivation [57]. These adipose tissue sources offer solutions to challenges posed by transgene delivery via AAVs, which may result in unintended effects on adjacent cell types or tissues. Estimations suggest that 50 grams of activated brown adipose tissue can potentially burn up to 500 calories daily, implying that the amount of material needed for transplantation may be relatively modest [58]. Moreover, subcutaneous transplantation offers the advantage of minimal invasiveness, with the possibility of repeated tissue replacement over time. While adipose tissue may offer certain advantages over the targeting other disease-related tissues with optogenetics technology, the challenges associated with its clinical translation remain significant and necessitate thorough consideration.

## Summary and Future Perspectives

The innate thermogenic capabilities and resistance to metabolic syndrome conferred by brown and beige adipose tissues make them suitable candidates for therapies for combating obesity and related metabolic disorders. Our understanding of brown and beige adipocyte signaling pathways, particularly UCP1-dependent and UCP1-independent mechanisms, sheds light on the potential for precise modulation of energy expenditure. The emergence of optogenetics as a revolutionary tool for cellular control adds an exciting dimension to this quest. Its ability to harness light-sensitive proteins for temporal and spatial control offers new avenues for manipulating brown and beige adipocytes. The studies highlighted here demonstrate the feasibility of inducing thermogenesis in these cells using optogenetic techniques, offering insights into signaling mechanisms and promising strategies for weight management and metabolic health. However, challenges remain, including optimizing the kinetics of brown adipocyte activation and the discovery of new, highly sensitive optogenetic proteins that can be used to sustain thermogenesis. These questions underscore the need for further research and refinement of optogenetic approaches. The future holds exciting possibilities as we continue to unlock the therapeutic potential of these remarkable tissues and cutting-edge optogenetics, however, more research is needed to translate this technology into clinical application. Overall, optogenetics offers a promising prospect of personalized interventions for individuals struggling with obesity while expanding our understanding of cellular regulation and energy balance in adipose tissues.

## Abbreviations:

WAT: White Adipose Tissue
BAT: Brown Adipose Tissue
UCP1: Uncoupling Protein-1
Ca2+: Calcium
ChR2: Channel Rhodopsin-2
LED: Light Emitting Diode
AAV: adeno-associated Virus
EYFP: Enhanced Yellow Fluorescent Protein
TH-Cre: Tyrosine Hydroxylase-driven Cre recombinase
β3-AR: β3-adrenergic Receptor
ATP: Adenosine Triphosphate
SERCA2: Sarco/endoplasmic Reticulum Ca2+-ATPase
HFD: High-fat Diet
ms: Milliseconds
Hz: Hertz
cAMP: Cyclic Adenosine Monophosphate
PKA: cAMP-dependent Protein Kinase A
CREB: cAMP Response Element-Binding Protein
AC: Adenylyl Cyclase
bPAC: Bacterial Photoactivatable Adenylyl Cyclase
BLUF: Blue Light sensor using Flavin adenine dinucleotide
ADSCs: Adipose-derived Stem Cells
iPSCs: Induced Pluripotent Stem Cells
